# Self-guided Positive Imagery Training: Effects beyond the Emotions–A Loreta Study

**DOI:** 10.3389/fnhum.2017.00644

**Published:** 2018-01-09

**Authors:** Svetla Velikova, Bente Nordtug

**Affiliations:** ^1^Central Scientific Research Laboratory, Nizhny Novgorod State Medical Academy, Nizhny Novgorod, Russia; ^2^Faculty of Nursing and Health Science, Nord University Bodø, Bodø, Norway

**Keywords:** positive imagery, cognition, EEG, connectivity, insula, depression, emotions, non-verbal reasoning

## Abstract

Previously we demonstrated that a 12-week lasting *self-guided positive imagery training* had a positive effect on the psycho-emotional state of healthy subjects and was associated with an increase in functional connectivity in the brain. Here we repeated the previous project, but expanded the study, testing the hypothesis that training can also affect cognitive functions. Twenty subjects (half of them with subthreshold depression according CES-D) participated in the program of *positive imagery training* for 12 weeks. The schedule began with group training for 2 days, followed by training at home. Evaluations of cognitive functions and electroencephalographic (EEG) activity were conducted during three examinations as follows: E_0_-baseline (1 month before the training); E_1_-pre-training and E_2_-post-training. CNS Vital Signs battery was used to test the following cognitive domains: verbal and visual memory, executive functions, cognitive flexibility, social acuity, non-verbal reasoning. EEGs (19-channel) were recorded at rest with closed eyes and analyzed with Low-resolution electromagnetic tomography software. One-way repeated measures ANOVA, followed by pairwise comparison showed a significant increase after training (E_2_ vs. E_1_; E_2_ vs. E_0_) in the number of *correct hits for positive emotions* received *during perception of emotions test (POET);* after the sample was split according to the initial presence of depressive symptoms, the effect was present only in the subgroup with subthreshold depressive symptomatology. Post-training (E_2_ vs. E_1_; E_2_ vs. E_0_) the number of correct answers on *non-verbal reasoning test* increased; this effect was observed only in the subgroup that does have any depressive symptoms. Comparison of EEG post-training vs. pre-training demonstrated a significant reduction in current source density (CSD) after the training in the left hemisphere (insular cortex, frontal and temporal lobes in delta, theta and alpha1 bands). The observed changes were presented only in the subgroup with initial subthreshold depressive symptomatology. A negative correlation was found between POET and CSD in the left insular cortex for theta band. No significant differences were observed when data from EEG and cognitive tests obtained during pre-training were compared with baseline values. Potential use of training for the rehabilitation of various disturbances with cognitive and emotional deficits is discussed.

## Introduction

The present work was devoted to the study of the effect of *self-guided positive imagery training* on the *cognitive domain*, and this was due to questions that arose during our pilot study on the abovementioned training methodology (Velikova et al., 2017). It was demonstrated that a 12-week long training improves the emotional well-being of healthy people, beneficially influencing their *mood* and subjective perception of *self-efficacy*. In addition, these subjective changes were accompanied by modifications of the cerebral *oscillatory activity* of participants revealed during the electroencephalographic (EEG) recording at rest, and this indicated that the training influences the functioning of the brain. In the mentioned study using Low Resolution Electromagnetic Tomography (LORETA) software (Pascual-Marqui, 2002), which allows 3-D reconstruction of the electrical density of the electrical sources of EEG signal, we investigated current source density (CSD) and LORETA based functional connectivity. There was an increase in the functional connectivity between the temporal regions from both hemispheres, measured by the lagged coherence, which was supposed to be due to improved coordination between the networks involved in image processing after training. The LORETA-based analysis revealed an increase in current source density (CSD) in the right Brodmann area 10 (BA10), which was hypothesized to be associated with observed emotional changes in participants.

Several studies report a link between EEG coherence at rest and some cognitive parameters (Anokhin et al., 1999; Thatcher et al., 2005; Chen et al., 2015; Hata et al., 2016). Furthermore, the practice of other mind-body techniques, such as meditation, has been found to increase functional connectivity (Fingelkurts et al., 2016) and to enhance the cognitive functions (Slagter et al., 2011; Saggar et al., 2012). The question then is whether emotional imagery exercises could have an impact that goes beyond influence on emotions: do they affect other cognitive functions? The oscillatory changes in BA10 observed in our previous study, can also be suggested as linked to eventual cognitive modulation, since this area is involved in many cognitive functions including working memory; the link between working memory and visual imagery being previously demonstrated (Likova, 2012). Finally, the fact of increased subjective self-efficacy again suggests possible changes in the cognitive field, since it has been shown that self-efficacy believes affect various aspects of human functioning, including cognitive processes (Bandura, 1994). On the other hand, it can't be ruled out that self-efficacy coefficients were influenced not only by mood changes, but also by improved cognitive performance.

Currently, the emotional imagery training (positive imagery) is recognized as a powerful tool in the psychotherapy of various emotional disturbances, and its ability to change some maladaptive cognitive schemes with healthier ones is proved (Holmes and Mathews, 2010; Pearson et al., 2015; Renner et al., 2017). However, *mapping the cross-points* between *emotions* and *cognition* in the context of *emotional imagery training* in healthy people, i.e., when the goal is “limited” to the optimization of affect, is a horizon that has not yet been explored, especially when the training is self-guided.

In this study, we tested the hypothesis that *self-guided positive imagery training* can affect cognitive functions beyond one's own emotions, so here, using a new group, we repeated our previous experiment (Velikova et al., 2017), expanding the study with a battery for cognitive evaluation.

Aside from testing a new hypothesis, and testing the reproducibility of the results obtained in the first study regarding the influence on mood and changes in brain oscillatory activity, the current study served to overcome the drawback of the previous study regarding the lack of a control group. Here we used the participants as controls to themselves.

## Materials and methods

### Subjects

Participants in the study were 20 volunteers (10 women and 10 men) aged 22–51 years (mean age 37.9 years). They were collected through an announcement in the social media (Facebook). Initially, semi-structured interviews with a psychiatrist were conducted to assess the psycho-emotional state and motivation of candidates. Excluding criteria were psychosis and affective disorders according to ICD-10 (ICD-10, World Health Organization, version 2010). Candidates with subthreshold depression were included. All the participants had “normal” life and social activity. They were free of medications and/or other medical interventions. The participants had no previous experience in mindfulness or meditation techniques and didn't have other types of mental training or psychotherapy during the observed period. Before the start of the study an informed written consent from all participants was obtained. This study was conducted in accordance with the Declaration of Helsinki.

### Training program

The training program was analogous with that described in our previous study (Velikova et al., 2017). In summary: the program duration was 12 weeks and included an initial 2-day long seminar with guided group training, followed by practice at home (15–20 min/daily) and then a *second* group training (2 days long) at the end of the observed period. The participants learned techniques in order to use imagery:

- To cope with the past psycho-traumatic events (through imagery transformation of a psycho-traumatic event to positive one);
- For goal achievement (through positive imagery of future events);
- To improve the social interactions (through positive imagery of social interactions and imagery of the emotions of other people);
- To enhance the emotional balance in daily life (participant learned to visualize the next day in a positive way).

Additionally, the participants were instructed to write a self-report on the regularity of the work performed. The sessions at home began with relaxation during countdown from 7 to 1, followed by imagery exercises thematically adapted to the current needs.

### Assessment schedule

Each assessment session consisted of an EEG record, a self-assessment of the emotional state on a paper basis, and a computerized evaluation of cognitive functions.

As in our previous study, an assessment was made before and after training. Furthermore, to establish the control condition, distinguish the possible “placebo” effect (in relation to evaluation of emotional state), and the effect of “learning” after repeating the task, a control assessment was held 1 month before the start of the training. So, the following three evaluations were made:

- E_0_ -baseline evaluation (1 month before the initial workshop)
- E_1_ -pre-training evaluation (up to 3 days before the initial workshop)
- E_2_ -post-training evaluation (up to 3 days after the completion of the training).

### Assessment

#### Self-evaluation

##### Tests description

For estimating the psycho-emotional changes, participants were asked to perform a self-assessment as follows:

- In order to determine the depression quotient, *the Center for epidemiologic studies depression (CES-D) 20 item scale* (Radloff, 1977) was used. CES-D is a self-report depression scale for research in the general population and measures the depressive feelings and behavior during the previous week.
- *Satisfaction with life scale (SWLS)* (Diener et al., 1985), developed as a measure of the judgmental component of subjective well-being.
- *General Self-Efficacy scale (GSE)* (Schwarzer and Jerusalem, 1995). The scale reflects the presence of optimistic self-confidence (Schwarzer, 1992), that is, the belief that one can perform a novelty or difficult task, or to cope with adversity in various domains.

##### Statistical analysis

The statistical analysis was performed using SPSS 13.0. For each test an one-way repeated measured analysis of variance (ANOVA) was conducted to evaluate the null hypothesis that there is no change in participants' scores when measured at the next time points: E0 (baseline evaluation), E1 (pre-training evaluation) and E2 (post-training evaluation). When ANOVA indicated significant main effect of time point, the analysis was followed by pairwise comparison applying Bonferroni correction. We used alpha level of.05 for all statistical tests.

#### Computerized assessment of cognitive functions

##### Tests

CNS-Vital Signs (CNSVS) battery was used for testing the next cognitive domains: verbal and visual memory, executive functions, processing speed, psychomotor speed, cognitive flexibility, social acuity and reasoning. For these purposes the following tests were selected: verbal and visual memory (VBM and VIM) tests, finger-tapping test (FTT), symbol digit coding test (SDC), Stroop test (ST), shifting attention test (SAT), perception of emotions test (POET), and non-verbal reasoning test (NVRT).

##### Statistical analysis

The obtained data from the scientific CNCVS rapports were analyzed using the same algorithm as described for the analysis of self–reports (see Statistical Analysis).

#### Electroencephalographic (EEG) recording and analysis

Electroencephalograms were obtained in all three assessment sessions (E_0_, E_1_, and E_2_) at rest with the eyes open and closed, each of them for duration of 5 min. Nineteen-channel EEGs were recorded with an “Encephalan” amplifier (Medicom MTD, Taganrog, Russia) using a cap with electrodes positioned according to the International 10/20 system (Jasper, 1958); linked ears were used as reference. The impedance of the EEG signal was below 5 kΩ; the sampling rate was 250 Hz. On-line filters were set up as follows: high-pass filter 0.5 Hz, low-pass filter 70 Hz and a notch filter at 50 Hz. The EEG data were visually inspected and artifact-free segments with ? length of 60 s were manually selected using NeuroGuide Deluxe (Applied Neuroscience Inc., Florida, USA) software version 2.8. In order to control for presence of drowsiness artifacts in the collected data during closed eyes, the segments were selected from the first 2 min of the EEG-recordings and had a test-retest reliability coefficient higher than 0.95. The test-retest reliability statistic is a good method to detect drowsiness when comparing the beginning of the EEG recording to the end of a lengthy recording with the eyes closed (Thatcher, 2010). It is considered that a coefficient higher than 0.9 indicates no dramatic change in the state of vigilance between the beginning and the end of the recording (Thatcher, 2010). Therefore, selecting segments from the initial period of the closed eyes state and high test-retest reliability coefficient minimizes substantially the probability of including drowsiness segments.

The study planned to compare the EEG obtained in eyes closed state, as this condition allows better research into possible changes in the alpha band and the default mode network that are of constant interest with regard to the practice of mind-body techniques (Fell et al., 2010; Fingelkurts et al., 2016). In addition, we recorded the EEG in the open eye state to compare alpha suppression on different records and, therefore, exclude the possibility that eventual EEG changes may result from different levels of relaxation. Originally alpha (8–12 Hz) power on occipital electrodes (O1 and O2) in both states of the open eyes and closed eyes were obtained using NeuroGuide. Then, for each participant, an “alpha suppression index” (calculated as the ratio between the alpha power in the open eye state and the closed eye state) was calculated. One-way repeated measures ANOVA did not find any significant differences in the alpha suppression index in different records. The analysis was then carried out using data obtained in the eyes closed condition.

The edited EEGs records were further analyzed using sLORETA software (Pascual-Marqui, 2002) version 20150415 (The KEY Institute for Brain-Mind Research, Zurich). LORETA resolves the inversion problem, maximizing the power of synchronization only between neighbor neuronal populations and allows 3-D reconstruction of the electrical density of the electrical sources. sLORETA performs source localization in 6239 cortical gray matter voxels sized 5 mm^3^. The solution space of sLORETA is defined via a reference brain from the Brain Imaging Center at the Montreal Neurological Institute (MNI). The software reports MNI coordinates (Jurcak et al., 2007). Anatomical labels such as Brodmann areas are reported using an appropriate correction from MNI to Talairach space (Brett et al., 2002). Therefore, sLORETA images represent the electric activity at each voxel in neuroanatomic Talairach space (Talairach and Tournoux, 1988) as the squared standardized magnitude of the estimated current density.

sLORETA's current source density in frequency domain (separate files for each person in each EEG assessment) was reconstructed on the basis of primary currents derived from EEG. For frequency domain the inverse solution (LORETA) is using discrete Fourier transform. The frequency bands were selected as follows: Delta (1.5–4 Hz), Theta (4.5–80 Hz), Alpha1 (8.5–10 Hz), Alpha2 (10.5–12 Hz), Beta1 (12.5–18 Hz), Beta2 (18.5–21 Hz), and Beta3 (21.5–30 Hz). The comparison of the distribution of CSD between the paired groups was implemented using non-parametric statistical analysis (Statistical non-Parametric Mapping; SnPM) employing the Log of ratio of averages (log of F-ratio) and performing SnPM randomization (number of randomizations = 5,000). The SnPM methodology corrects for multiple comparisons and does not require gaussianity assumptions (Nichols and Holmes, 2002).

## Results

### Self-evaluation tests

The results of one-way repeated measures ANOVA indicated a significant main effect of the time point of measuring on the performance for all three self-evaluation scales (CES-D, SWLS and GSE). The mean values with their standard deviations and indications for pairs with significant differences are presented in Table 1.

**Table 1:** On the table are presented the mean values with standard deviations for performance on: center for epidemiologic studies depression (CES-D), Satisfaction with life scale (SWLS), General Self-Efficacy scale (GSE), number of correct responses in Non-verbal reasoning test (NVRT) and correct hits for positive emotions in Perception of Emotions Test (POET).

		**Group (*n* = 20)**	**Subgroup with CES-D < 16 (*n* = 10)**	**Subgroup with CES-D > 16 (*n* = 10)**
CES-D	E_0_	$\begin{array}{l} 18.35\pm 10.97\\ \begin{array}{l} 17.8\pm 11.86\\ 8.10\pm 9.64 \end{array}\}* \end{array}\}*$	9.0 ± 4.92 9.2 ± 4.73 6.4 ± 11.05	$\begin{array}{l} 27.7\pm 5.96\\ \begin{array}{l} 26.4\pm 6.11\\ 9.8\pm 8.23 \end{array}\}* \end{array}\}*$
	E_1_			
	E_2_			
SWL	E_0_	$\begin{array}{l} 21.45\pm 5.61\\ \begin{array}{l} 21.4\pm 6.464\\ 25025\pm 7.03 \end{array}\}* \end{array}\}*$	25 ± 3.89 24.5 ± 4.25 27.4 ± 6.19	$\begin{array}{l} 17.9\pm 4.82\\ \begin{array}{l} 17.7\pm 6.67\\ 23.1\pm 7.48 \end{array}\}* \end{array}\}*$
	E_1_			
	E_2_			
GSE	E_0_	$\begin{array}{l} 30.2\pm 4.3\\ \begin{array}{l} 31.1\pm 4.8\\ 33.7\pm 4.82 \end{array}\}* \end{array}\}*$	$\begin{array}{l} 31.7\pm 4.19\\ \begin{array}{l} 33.2\pm 3.8\\ 34.4\pm 4.83 \end{array}\}* \end{array}\}*$	$\begin{array}{l} 28.7\pm 4.06\\ \begin{array}{l} 29\pm 4.94\\ 33\pm 4.97 \end{array}\}* \end{array}\}*$
	E_1_			
	E_2_			
NVRT correct responses	E_0_	$\begin{array}{l} 10.15\pm 1.42\\ \begin{array}{l} 10.15\pm 1.46\\ 11\pm 1.56 \end{array}\}* \end{array}\}*$	$\begin{array}{l} 10.5\pm 1.43\\ \begin{array}{l} 10.2\pm 1.45\\ 11.6\pm 1.17 \end{array}\}* \end{array}\}*$	9.8 ± 1.4 10.1 ± 1.37 10.4 ± 1.65
	E_1_			
	E_2_			
POET Correct hits pos.emotions	E_0_	$\begin{array}{l} 5.2\pm 0.83\\ \begin{array}{l} 5.45\pm 0.76\\ 5.75\pm 0.55 \end{array}\}* \end{array}\}*$	5.4 ± 0.7 5.8 ± 0.52 5.7 ± 0.42	$\begin{array}{l} 5\pm 0.88\\ \begin{array}{l} 5.1\pm 0.74\\ 5.8\pm 0.32 \end{array}\}* \end{array}\}*$
	E_1_			
	E_2_			

*The data are reported for the next examinations: E_0_-baseline (1 month before start of the training); E_1_ - pre-training (up to 3 days before start); E_2_ post –training (up to 3 days after finishing to training period). With asterisk are indicated the pairs of values which are significantly different*.

#### Center for epidemiologic studies -depression scale (CES-D)

Initial assessment with CES-D showed that 10 participants had subthreshold depression (score higher than 16, which represents cutoff for “non-significant” or “mild” depressive symptomatology (Radloff, 1977), but they did not meet the ICD-10 criteria for depression). At the end of the training, according to CES-D, participants had less prominent depressive symptomatology than at baseline and the number of those having a score of more than 16 was reduced to two (none of them met the criteria for depression). To take into account that half of the group had initially subthreshold depressive symptomatology, an additional analysis was made, dividing the group into two subgroups by the initial score on CES-D (CES-D < 16 and CES-D > 16).

#### CES-D -comparison applied for the whole group

One-way repeated measures ANOVA indicated a significant main effect of time of evaluation on CES-D performance [Wilks'Lambda = 0.548, *F*_(2, 18)_ = 7.414, *p* = 0.004, η^2^ = 0.731]. There is significant evidence thus, to reject the null hypothesis. Pairwise comparison adjusted for multiple comparison (Bonferroni), indicated significant (*p* = 0.047) decrease in the CES-D scores measured post-training (E2) in comparison with pre-training (E1). Post-training (E2) obtained CES-*D*-values also were significantly (*p* = 0.03) lower in comparison with the values from the baseline (E0). The comparison of CES-D values obtained during pre-training examination (E1) with values from baseline (E0) showed no significant changes. This supports the hypothesis that time of the assessment affects participants' self-perception of own depressing feelings and behavior during the previous week. These findings suggest that after 12 weeks of training the participants had fewer depressive feelings.

#### CES-D-comparison applied to subgroups

After the splitting of the group, a significant main effect of time on CES-D scores was found in the subgroup with subthreshold depressive symptomatology [Wilks'Lambda = 0.074, F_(2, 8)_ = 50.028, p < 0.001] but not in the other half of the group. The pairwise comparison of CES-D values obtained pre-training (E1) and post-training (E2) showed a significant (p < 0.001) decrease of CES-D post-training (E2). The CES-D values from post-training measurement (E2) also were significantly (p < 0.001) lower when compared with values form the baseline (E0). No significant difference was found when pre-training (E1) and baseline (E0) values of CES-D were compared.

#### Satisfaction with life scale (SWLS)

##### SWLS-comparison applied for the whole group

One-way repeated measures ANOVA indicated a significant main effect of time of evaluation on the satisfaction with life as perceived by the participants [Wilks'Lambda = 0.626, *F*_(2, 18)_ = 7.414, *p* = 0.015, η^2^ = 0.374]. The following pairwise comparisons (Bonferroni corrected) indicated a significant (*p* = 0.01) increase in SWLS values obtained post-training (E2) in comparison with pre-training (E1) values. The post-training (E2) values also were significantly (*p* = 0.014) higher in comparison with baseline (E0) scores. There was no significant difference in SWLS scores when pre-training (E1) and baseline (E0) values were compared. These findings indicate that after the training, the participants rated themselves as more satisfied with their lives.

##### SWLS-comparison applied to subgroups

After the splitting of the group, a significant main effect of time on SWLS was found only in the subgroup with subthreshold depressive symptomatology [Wilks'Lambda = 0.395, *F*_(2, 8)_ = 6.135, *p* = 0.024]. Consecutive pairwise comparisons showed that SWLS estimates obtained at post-training evaluation (E2) were significantly (*p* = 0.015) higher in comparison with the values at baseline (E0). Post-training values also were significantly (*p* = 0.027) higher than pre-training (E1) values. Analysis showed no significant difference between the estimates obtained during pre-training (E1) and baseline (E0) evaluations.

#### General self-efficacy scale (GSE)

##### Comparison applied for the whole group

One-way repeated measures ANOVA showed a significant main effect of time of evaluation on perceived self-efficacy [Wilks'Lambda = 0.462, *F*_(2, 18)_ = 10.487, *p* = 0.001, η^2^ = 0.538]. The following pairwise comparisons (Bonferroni corrected) indicated that post-training values (E_2_) were significantly (*p* = 0.019) higher in comparison with pre-training (E_1_) scores. The post-training (E_2_) values also were significantly (*p* = 0.001) higher than baseline (E_0_) values. No significant difference was found between GSE measured during pre-training (E_1_) and baseline evaluations (E_0_).

##### GSE- comparison applied to subgroups

After the splitting of the group, a significant main effect of time on GSE was detected for both subgroups. For the subgroup without depressive tendencies (CES-D < 16), ANOVA found [Wilks'Lambda = 0.259, *F*_(2, 8)_ = 11.459, *p* = 0.004]. The pairwise comparison showed that post-training (E_2_) values were significantly (*p* = 0.03) higher than at baseline (E_0_). For the subgroup with subthreshold depressive symptomatology: [Wilks'Lambda = 0.3, *F*_(2, 8)_ = 8.915, *p* = 0.009]. The pairwise comparison showed that post-training (E_2_) values were significantly (*p* = 0.02) higher than at baseline (E_0_). Post-training (E_2_) scores also were significantly (*p* = 0.006) higher in comparison with pre-training (E_1_) values; no difference was observed between *GSE* values obtained during pre-training (E_1_) and baseline (E_0_) evaluations.

### Cognitive tests (CNS vital signs)

One-way repeated measures ANOVA indicated a significant main effect of time of evaluation on results obtained in Non-verbal reasoning test (NVRT)- section Number of correct responses and Perception of Emotions Test (PET)- section Correct hits for positive emotions.

#### Non-verbal reasoning test (NVRT)

##### NVRT-comparison applied for the whole group

For *Number of correct responses* on *Non-verbal reasoning test (NVRT-cr)* ANOVA indicated Wilks'Lambda = 0.616, *F*_(2, 18)_ = 5.612, *p* = 0.013,η^2^ = 0.384. The following pairwise comparison (Bonferroni corrected) showed a significant (*p* = 0.014) increase in *NVRT-cr* values measured at post-training (E_2_) evaluation when compared with pre-training (E_1_) values. Post-training (E_2_) values also were significantly (*p* = 0.027) higher than at baseline (E_0_). No significant difference was found between *NVRT-cr* values obtained during pre-training (E_1_) evaluation and baseline (E_0_).

##### NVRT comparison applied to subgroups

After the splitting of the group, a significant main effect of time on *NVRT-cr* was found only for the subgroup without depressive tendencies [Wilks'Lambda = 0.221, *F*_(2, 8)_ = 14.116, *p* = 0.002]. The pairwise comparison showed significantly (*p* = 0.001) enhanced post-training (E_2_) in comparison with pre-training (E_1_) scores. Post-training (E_2_) values also were significantly (*p* = 0.02) higher than at baseline (E_0_). No significant difference was found between *NVRT-cr* values obtained pre-training (E_1_) when compared with baseline (E_0_).

#### Perception of emotions test (POET)

##### POET comparison applied for the whole group

For Correct hits for positive emotions on Perception of Emotions Test (POET-chpe) ANOVA indicated Wilks'Lambda = 0.534, *F*_(2, 18)_ = 7.859, *p* = 0.004, η^2^ = 0.466. The following pairwise comparison (Bonferroni corrected) demonstrated significant (*p* = 0.030) increase in the number of POET-chpe post-training (E2) when compared with pre-training (E1); The number of POET-chpe obtained during post-training (E2) evaluation was significantly (*p* = 0.006) higher than at baseline (E0). No significant difference was found when compared POET-chpe values measured pre-training. (E1) vs. baseline (E0).

##### POET comparison applied to subgroups

After the splitting of the group, a significant main effect of time on POET-chpe was found only for the subgroup with subthreshold depression [Wilks'Lambda = 0.421, *F*_(2, 8)_ = 5.495, *p* = 0.031]. The followed pairwise comparison showed significantly (*p* = 0.032) enhanced values in POET-chp performance, when compared post-training (E2) vs. pre-training (E1); post-training (E2) values also were significantly (*p* = 0.025) higher in comparison with baseline (E0); no significant differences were observed between pre-training (E1) and baseline (E0).

### EEG analysis

#### sLORETA comparison applied for the whole group

**Figure 1:**
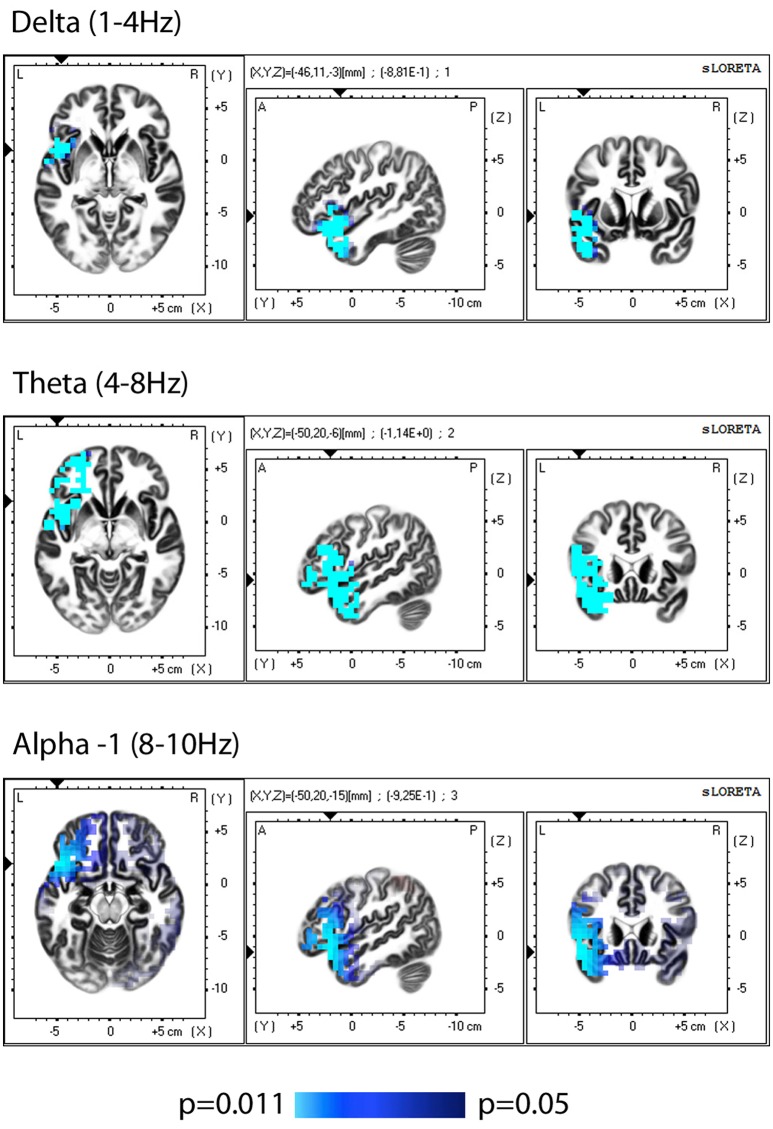
sLORETA group comparison of the current source density(CSD) before and after training, employing Log of the F-ratio statistic and SnPM randomization. The voxels with significant (*p* ≤ 0.05) decrease in CSD are presented in bleu.

sLORETA-based (Low resolution electromagnetic tomography) comparison of EEGs (eyes closed) before training vs. baseline showed no significant differences. The comparison of the data obtained after training vs. pre-training, demonstrated a significant reduction in the current source density after the completion of the training period in the left hemisphere (insular cortex, frontal, and temporal lobes), observed in the delta, theta and alpha-1 bands (Figure 1, Table 2).

**Table 2:** Regions with a significantly decreased current source density after training, described by Brodmann area, lobe, gyrus, and oscillatory band, where changes are observed.

**Brodmann area, lobe, gyrus**	**Delta 1.5–4.0 Hz**	**Theta 4.5–8 Hz**	**Alpha-1 8.5–10 Hz**
38 left, Temporal Lobe, Superior Temporal Gyrus	✓ ☑	✓ ☑	✓ ☑
22 left, Temporal Lobe, Superior Temporal Gyrus	✓ ☑	✓ ☑	
21 left, Temporal Lobe, Middle Temporal Gyrus	✓ ☑	✓ ☑	
20, Temporal Lobe, Inferior Temporal Gyrus	☑	✓	
47 left, Frontal Lobe, Inferior Frontal Gyrus	✓ ☑	✓ ☑	✓ ☑
46 left, Frontal Lobe, Inferior Frontal Gyrus		✓	
45 left, Frontal Lobe, Inferior Frontal Gyrus	☑	✓ ☑	✓
44 left, Frontal Lobe, Precentral Gyrus	☑	✓ ☑	
11 left, Frontal Lobe, Middle Frontal Gyrus	☑	✓ ☑	
10 left, Frontal Lobe, Inferior Frontal Gyrus	☑	✓ ☑	
9 left, Frontal Lobe, Inferior Frontal Gyrus	☑	✓ ☑	
6 left, Frontal Lobe, Precentral Gyrus	☑	✓ ☑	
13 left, Sub-lobar, Insula	☑	✓ ☑	
34 left, Limbic Lobe, Parahippocampal Gyrus	✓	✓	
28 left, Limbic Lobe, Uncus		✓	

*With “✓” is marked the differences observed in the entire (N = 20) group; with “☑ are presented the differences observed in the subgroup (n = 10) with subthreshold depressive symptomatology*.

http://www.cnsvs.com

http://www.medicom-mtd.com/en/index.html

http://www.appliedneuroscience.com

http://www.uzh.ch/keyinst

#### sLORETA comparison applied to subgroups

After the sample was divided sLORETA analysis showed significant difference (decreased CSD) after training only in the group with subthreshold depressive symptomatology, and the distribution of the changes according to the regions and frequency bands almost coincided with the observed group effect.

### Correlation analysis

#### Correlation analysis applied for the whole group

**Figure 2:**
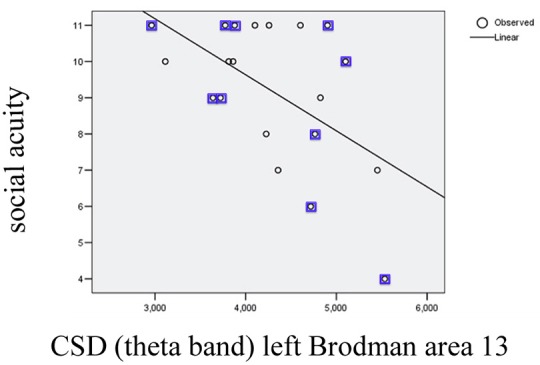
Scatterplot for correlation between social acuity assessed by perception of emotions test and current source density (CSD) in left Brodmann area 13 measured in theta band (*r*^2^ = 0.303). The values indicated with squares belong to the subgroup with initial subtreshold depressive symptomatology.

*I*n order to investigate possible correlations between cognitive performance and EEG parameters post-training, values from the cognitive tests were subjected to partial correlation analysis (using as control factors *gender* and *age*) with CSD in the areas showing significant changes after training (the list corresponds to the areas/bands described on Table 1). The analysis (SPSS.13) demonstrated a negative partial correlation between the *Social acuity* measured by perception of emotions test and current source density in the left Insular cortex (Brodmann area 13) for the theta band (*r* = −0.494, *p* = 0.037); the scatterplot for observed correlation is presented in Figure 2.

#### Correlation analysis applied to subgroups

After splitting the group no significant correlation was found on the level of subgroups. No other correlation between cognitive performances and EEG parameters measured post-training were observed.

## Discussion

### Self-evaluation tests

This work confirmed the results obtained in our previous study (Velikova et al., 2017) that self-guided positive imagery training, implemented in accordance with the protocol described, had a positive impact on the emotional state of participants, they were more satisfied with their lives, and they perceived themselves as more effective. For all tests no significant differences were observed between the performances during baseline (E_0_) and pre-training (E_1_) examinations, therefore the effects should not be explained by the repetition of the tasks.

The results show that basically the group with subthreshold depressive symptomatology led to a group effect for improvement on the CES-D scale and on life satisfaction scale, while improvement in the scale of overall General Self-Efficacy was found in both subgroups.

### Cognitive tests

The study confirmed the initial hypothesis that the training can influence cognitive functions. It was found that after the training participants had enhanced their performance with respect to perception of emotions and had better results on non-verbal reasoning test. The improvement on “perception of emotions test” was mainly derived by the changes observed in the subgroup with subthreshold depression. The finding that the “perception of emotions” has improved along with the reduction of depressive symptoms can be seen in accordance with the fact that during a depressive episode the ability to recognize faces as happy decreases and, conversely, improves with decrease in depressive symptoms (Münkler et al., 2015).

The non-verbal reasoning was a unique domain where significant changes were observed only in half of the sample without depressive symptomatology. A possible explanation may be consistent with the study (Takesaki et al., 2016), demonstrating a positive relationship between functional connectivity and non-verbal reasoning, and although we did not study functional connectivity here, our previous study of emotional images (Velikova et al., 2017) demonstrated an increased communication after training. On the other hand it known that capacity to imagine is reduced in presence of depressive symptomatology (Hackmann et al., 2011). Taken together, we assume that the difference in performance on non-verbal reasoning test is due to the different ability to imagine, which consequently leads to less changes in the connectivity, therefore lower performance.

### Electroencephalographic data

The present study affirmed our previous data that training influenced the activity of the brain at rest. Comparison of the EEG data post-training vs. pre-training using sLORETA, showed a decrease in the CSD for the low-frequency bands (delta, theta, alpha-1) in the left hemisphere, which included the insular cortex, frontal, temporal, and limbic lobes. Analysis after split of group showed that the observed changes at the group level were mainly related to changes in the half of the sample with subthreshold depression.

Some of the observed regional changes can be viewed as related to emotional and cognitive changes.

Insular cortex is involved in regulation of emotions (Craig, 2011), in addition, it is viewed as as a cross point for both emotional and cognitive processes (Kurth et al., 2010), so the change in the CSD in this region could be seen in regard to these functions. The observed lateralization of changes in the *left* insular cortex can be considered associated with the type of training. As discussed by (Gu et al., 2013), the main hypothesis regarding the function of the *left* (anterior) insular cortex suggests that, firstly, it directly processes both positive and negative feelings, and secondly, it specifically encodes “energy-nourishing” positive feelings for difference with the right insular cortex, which encodes “energy-consuming” negative feelings (Craig, 2011). Thus, the reinforcement of positive emotions, as might be expected, will affect the activity of the left insular cortex. Our results can also be seen in accordance with the results presented by Baldwin et al. (2011), indicating that greater left hemispheric activation was associated with a more positive psychological profile. The data obtained here on the reduction in CSD in the insular cortex in parallel with the positive emotional changes in participants can be interpreted in accordance with data indicating an opposite relationship: an increase in CSD in the insular cortex in anxiety disorders (Velikova et al., 2010).

Insular cortex also plays a role in sensitivity to the emotions of others (Ruiz et al., 2013; Terasawa et al., 2015), and observed here relationship between the index of social acuity (POET) and CSD for theta band in the left insular cortex can be hypothesized associated with this role. Furthermore, the insular cortex, along with the prefrontal cortex, where functional modifications also were found, is included in the network associated with *social cognition* (Watanabe et al., 2014).

Observed here changes in the functions of the prefrontal cortex after training can be seen in accordance with the results of our previous study on the same technique, where also was observed changes in CSD in prefrontal cortex (Velikova et al., 2017). We assume that the changes in BA10 may be related to his role in imagining pleasant scenes (Costa et al., 2010), and self-reflection (Johnson et al., 2002), whose practice was part of the training. In addition, BA10 participates in the regulation of emotions (Liotti et al., 2002), and also contributes to the degree of life satisfaction (Kong et al., 2015), parameters that have been significantly altered after training.

Changes in temporal regions also were found in our previous study and interpreted as probably related to image elements in the exercises, since the temporal regions are involved in the image processing (Mellet et al., 1996). Another interpretation in the same direction could be that observed changes in temporal regions are part of the changes in the network modulated during the practice of mind-body techniques in general, since changes in the same network as described here (including temporal regions) were found in experienced mediators at rest (Brewer et al., 2011).

The frequency range of the EEG changes observed here was mainly in theta and less in the delta and alpha1 ranges, which is consistent with the results of our previous study. Theta are the most consistently reported frequencies linked to creative processing (Petsche, 1996), and since training was composed of several creative elements, this can be a possible direction for interpreting the theta changes.

Since EEG changes have been observed at low frequencies, the question is whether they can be associated with a change in the level of vigilance? We believe that this is implausible, since: (1) the initial EEG was precisely controlled to eliminate drowsiness segments using a high test-retest reliability index; (2) the observed changes in the EEG are focal, affecting only one hemisphere, which does not correspond to the topography of possible changes in drowsiness.

On the other hand, changes in low frequencies, including unilateral changes, have been previously reported in connection with the practice of various mind-body techniques (Berkovich-Ohana et al., 2014). The changes observed here in delta and theta bands also confirm the results of our previous study, where we reported changes in delta and theta connectivity after the same training (Velikova et al., 2017).

In addition to the similarities observed in the current study, EEG changes differ from those found in the first study, which may be the result of several factors, such as: two groups are different, and it can be assumed that a priori that the participants in the groups have different EEG-profiles, they can differ depending on the ability to imagine, unequal motivation and effort during training, etc.

#### Perspectives for practical application

In the first report on *self-guided positive imagery training*, we emphasized the impact of training on the emotional state, while this experiment also was expanded to study the effect on cognition. In addition to a beneficial impact on emotional well-being, the potential for increasing the effectiveness of non-verbal reasoning can be a matter of practical interest for use in both pathological states and among healthy subjects.

Training could be useful in neuro-rehabilitation for pathological conditions with disturbance in non-verbal reasoning, such as traumatic brain injury (Livny et al., 2016), and such patients can benefit from improving emotional well-being. The ability to improve emotions, interpersonal relationships and reasoning abilities presupposes a possible benefit from the application of training for academic purposes. Finally, non-verbal reasoning also is of great importance for daily life, so the method may be appropriate for inclusion in self-development programs.

## Ethics statement

The project was reviewed and approved by Regional Ethics Committee South East Norway (D 2016/921). The project was approved by NSD-Data Protection Official for research (48442).

## Author contributions

SV recorded EEGs, made EEG analysis and wrote the manuscript; BN analyzed the data from cognitive tests; both authors participated in planning the experiment, discussing the results and approving the manuscript.

### Conflict of interest statement

The authors declare that the research was conducted in the absence of any commercial or financial relationships that could be construed as a potential conflict of interest.
